# Research on Strengthening Fragile Paper with Polyvinylamine

**DOI:** 10.3390/polym16050619

**Published:** 2024-02-24

**Authors:** Jing Li, Meirong Shi, Yuhu Li, Peng Fu

**Affiliations:** 1 Shandong Museum, Jinan 250014, China; lijing9669@126.com; 2 Shaanxi Institute for the Preservation of Culture Heritage, Xi’an 710075, China; shimeirong123@163.com; 3 Engineering Research Center of Historical Cultural Heritage Conservation, Ministry of Education, School of Materials Science and Engineering, Shaanxi Normal University, Xi’an 710119, China

**Keywords:** polyvinylamine, paper documents, fragile paper, paper reinforcement

## Abstract

Paper documents are an important carrier of information related to human civilization, with the reinforcement and protection of fragile paper documents being a key aspect of their protection. This research utilized amphoteric polyvinylamine polymer as a paper reinforcement agent, strengthening the Xuan paper commonly used in paper documents. Scanning electron microscopy (SEM), Fourier transform infrared spectroscopy (FTIR), X-ray diffraction spectroscopy (XRD), X-ray photoelectron spectroscopy (XPS), solid-state 13C NMR, and other analytical methods were employed to compare the physical properties, micro-morphology, crystallinity, and aging resistance of the paper before and after reinforcement. Research was conducted on the effects of reinforcement, the aging resistance, and the effects on the fiber structure. The results indicate that polyethylenimine has a filling and bridging effect between the paper fibers. After treatment with polyethylenimine, there was a significant improvement in the folding endurance and tensile strength of the paper. Additionally, the paper maintains a good mechanical strength even after undergoing dry heat and humid aging.

## 1. Introduction

Paper literature is an important carrier of material related to human civilization, carrying information from various aspects such as history, society, culture, etc. It is a precious and nonrenewable heritage. The prolonged safeguarding of paper literature is attributed to elements like light [1], temperature, humidity, microorganisms, and acidic gases [2]. The cellulose in paper is prone to hydrolysis and oxidation, resulting in a reduction in the mechanical properties of the paper, fracture, and damage. Strengthening fragile paper and extending its durability are crucial elements in paper literature protection.

At present, the materials used for reinforcing paper literature, both domestically and internationally, mainly include natural polymer compounds and synthetic polymer compounds. Natural polymer compounds include starch [3,4], chitosan [5,6,7], gelatin [8,9,10], guar gum [11], nanocellulose, and bacterial cellulose. Owing to their molecular structure and properties that are similar to paper, they have emerged as a significant research direction for paper literature restoration. Organic compounds such as polyurethane, latex, acrylamide [12], and amino silane coupling agents [13,14] constitute synthetic polymer compounds. They mainly bind to hydroxyl groups in cellulose through crosslinking or chemical grafting [15,16]. The chemical bonds formed through crosslinking or grafting have a higher bond energy, a better mechanical strength [17], and stronger resistance to external damage [18].

Polyvinylamine (PVAm) is a type of straight-chain cationic or amphoteric water-soluble polymer whose amino groups are directly connected to a hydrocarbon framework. Its attributes include a small particle size, the ability to form films easily, and a stable performance, rendering it a promising candidate for diverse applications in sectors such as papermaking, biomedicine, and dyes. Rasteiro et al. [19] investigated the relationship between flocculation, drainage, and retention of cationic polyvinylamine in the papermaking process. Geffroy et al. [20] investigated the adsorption mechanism of cationic polyvinylamine on fibers. Lee et al. [21] utilized polyelectrolyte multilayer (PEM) technology to prepare antibacterial paper using polyvinylamine, observing a significant reduction in the proportion of bacteria on the surface of treated paper. Yang et al. [22] investigated the mechanism by which polyvinylamine enhances the wet adhesion between cellulose surfaces.

The method of reinforcing fragile paper with polyethylenimine demonstrates remarkable novelty and innovation. This technique utilizes the organic polymer material polyethylenimine to coat the paper surface and form interconnections with cellulose, thereby enhancing the mechanical strength and durability of the paper. This approach not only avoids potential damage caused by traditional reinforcement methods such as glue or tape, but also offers noticeable advantages in terms of practicality and environmental friendliness, providing effective safeguarding for valuable literature and historical archives. In this study, various analytical and testing methods, including scanning electron microscopy (SEM), Fourier transform infrared spectroscopy (FTIR), X-ray diffraction (XRD), X-ray photoelectron spectroscopy (XPS), and solid 13C NMR nuclear magnetic resonance, were employed to compare the physical properties, microstructure, crystallinity, thermal stability, and resistance to aging before and after paper reinforcement. The objective was to investigate the effectiveness of the reinforcement, aging resistance, and the impact on fiber structure, aiming to provide a novel approach and new perspectives for reinforcing and preserving paper-based literature.

## 2. Experimental Section

### 2.1. Experimental Materials

Polyethylenimine (Mitsubishi Chemical Co., Ltd., Tokyo, Japan) with a molecular weight of 800,000 was used. Potassium aluminum sulfate (analytically pure, Tianjin Damao Chemical Reagent Factory, Tianjin, China) commonly used in the production of paper and pulp to enhance water retention and fiber strength was used. Raw Xuan paper (“Wangtonghe” rice paper factory in Jing County, Anhui Province, China) is a traditional paper made from straw, renowned for its distinctive texture and strength. Due to its natural aesthetic and practical properties, it is frequently employed in calligraphy, printing, and packaging applications.

### 2.2. Sample Preparation and Processing

#### 2.2.1. Simulated Paper Sample Preparation

In this study, raw Xuan paper (handmade paper, mainly made of sandalwood bark and rice straw) was chosen as the paper sample. A 3% alum solution was prepared by mass fraction, it was applied evenly onto raw rice paper with a soft bristled brush and air-dried naturally, and a simulated paper sample was prepared. The PVAm loading per square centimeter of paper was 22 mg, and the mass ratio of paper to PVAm per unit area was 0.12.

#### 2.2.2. Paper Reinforcement Treatment

A solution of polyethylenimine (PVAm) was prepared by dissolving a polyethylenimine agent with a molecular weight of 800,000 in water as the solvent, resulting in a 4% PVAm solution. The prepared simulated paper sample, made of raw Xuan paper, was placed flat on a clean platform. The PVAm solution was uniformly applied to the paper sample using a soft bristle brush, with an application rate of approximately 0.5 mL/cm^2^. The solution penetrated the paper through the combined action of capillary effects and diffusion. Finally, the paper sample was left to air dry naturally.

#### 2.2.3. Aging Experiment of Paper

Both dry and wet heat aging were performed on untreated simulated paper samples and paper samples reinforced with a 4% mass fraction of PVAm solution. The paper samples were cut to a length of 250 mm and a width of 15 mm ± 0.1 mm. Dry heat aging: According to the national standard GB/T 464-2008 [23] “Dry heat accelerated aging of paper and paperboard”, the paper samples before and after reinforcement were placed in a dry heat aging test chamber and maintained at 105 °C for a duration of 72 h. Wet heat aging: According to the national standard GB/T 22894-2008 [24] “Wet heat treatment of accelerated aging of paper and paperboard under 80 °C and 65% relative humidity conditions”, the paper samples before and after reinforcement were placed in a wet heat aging test box and maintained at 80 °C and a relative humidity of 65%, with an aging time of 72 h.

### 2.3. Experimental Methods

#### 2.3.1. Color Difference Test

An X-Rite VS450 non-contact spectrophotometer was employed to test the color change of the paper before and after reinforcement. The CIE L*a*b* color space coordinate system was used to characterize the color change (color difference value) of the paper sample. The larger the color difference value ΔE, the more obvious the color change of the paper sample.

#### 2.3.2. pH Test

According to the national standard GB/T 1545-2008 [25] “Determination of acidity or alkalinity of water extracts from paper, paperboard and pulp”, 2.0 g of a paper sample was weighed and cut into 5–10 mm^2^ fragments. The sample was placed in a clean beaker, covered with 100 mL of distilled water, waiting for the paper to fully moisten, then it was sealed with cling film and left undisturbed for 1 h. The beaker was shaken 1–2 times throughout this duration. After 1 h, the solution was poured into a clean small beaker and the pH value of the extraction solution was measured using a METTLER TOLEDO FE28 pH meter (Mettler Toledo Technology (China) Co., LTD, Shanghai, China).

#### 2.3.3. Mechanical Performance Testing

Referring to the national standards GB/T 457-2008 [26] “Determination of Folding Resistance of Paper and Boardboard” and GB/T 12914-2008 [27] “Determination of Tensile Strength of Paper and Boardboard”, a folding tester and a paper tensile machine were employed to test the paper’s folding resistance and tensile strength. The sample was placed vertically between two clamps, with a gauge distance of 10 cm and a stretching rate of 20 mm/min. The paper was stretched until it broke to obtain the tensile strength S (kN/m). The tensile strength test was conducted horizontally and vertically. Using an MIT folding tester, the paper’s folding resistance was tested under a tension of 4.9 N, measured by the number of double folds. The average of 20 sets of data was taken for each paper sample.

#### 2.3.4. Microscopic Morphology

A paper sample of appropriate size was fixed flat onto the sample stage of a scanning electron microscope with conductive adhesive, and gold was sprayed on the surface of the sample to improve its conductivity. To observe the microstructure of the paper, an SU3500 tungsten filament scanning electron microscope in high vacuum mode with an accelerated voltage of 5 kV was employed in the experiment.

#### 2.3.5. FTIR

A Nicolet iS50 (Thermo Fisher Technologies, Waltham, MA, USA) Fourier transform infrared spectrometer was employed to examine paper samples before and after reinforcement treatment using a polyethylene amine solution. The KBr compression transmission technique was employed, involving 4 scans and a scanning range of 450–4000 cm^−1^.

#### 2.3.6. XRD

The paper was positioned on the sample stage and an X’Pert3 Powder X-ray diffractometer (PANalytical B.V., Almelo, Netherlands) was employed to examine the paper’s diffraction curves before and after treatment. The intensity of both diffraction peaks was compared and the alternations in the fiber crystal structure were analyzed. The test conditions were as follows: target type, Cu; radiation, Cu-Kα; voltage, 40 kV; current, 40 mA; testing range, 2θ. The scanning angle was 10~50, and step scanning was used with a scanning step size of 0.013° and a step scanning time of 8.67 s.

#### 2.3.7. XPS

An ESCALAB Xi^+^ X-ray photoelectron spectrometer (Thermo Fisher) was used to perform XPS testing on paper, both prior to and following the application of the polyethylene amine solution. The X-ray beam spot was 650 μm. The range of energy scanning spanned from 0 to 1300 eV, with a passing energy of 100 eV.

#### 2.3.8. Solid 13C NMR Test

A JNM-ECZ400R/S1 NMR spectrometer (JEOL Japan Electronics Co., Ltd, Tokyo, Japan) was used to perform 13C NMR testing on paper, both prior to and following treatment with the polyethylene amine solution. The probe diameter was 7 mm, the field strength was 7.05 T, the pulse width was 90°, the rotational speed was 12 kHz, and the cross polarization time was 4 μs, with a sampling interval of 2.5 s and a contact time of 800 μs.

## 3. Results and Discussion

### 3.1. The Influence of Polyethylene Amine on the Physical Properties of Paper

After testing, the paper samples exhibited pH levels of 4.73 and 7.24 before and after polyethylene amine treatment, respectively, and the color difference value was 0.92, signifying a shift from acidic to neutral in the treated paper, while the color change of the paper was insignificant.

**Figure 1:**
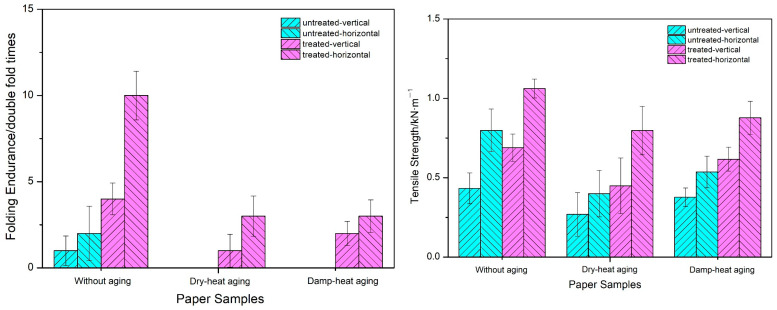
Folding endurance and tensile strength of paper samples before and after PVAm treatment.

The tensile strength and folding resistance of paper samples before and after polyethylene amine treatment are depicted in Figure 1. Observations reveal that the untreated paper has lower folding and tensile strengths. After aging, the folding and tensile strengths of the paper decreased by varying degrees, rendering its folding strength unmeasurable following both the dry heat aging and wet heat aging processes. The reductions in the folding and tensile strengths of the paper after dry heat aging are greater than those after wet heat aging, indicating that the aging of the paper after dry heat aging is more severe. After polyethylene amine treatment, there was an enhancement in both the paper’s resistance to folding and its tensile strength. Under the same aging conditions, the degree of reduction in the folding endurance and tensile strength before PVAm treatment is greater than that after treatment. This indicates that the paper treated with PVAm maintains its mechanical properties well and its aging resistance is enhanced.

### 3.2. The Effect of Polyethylene Amine on the Microstructure of Paper

**Figure 2:**
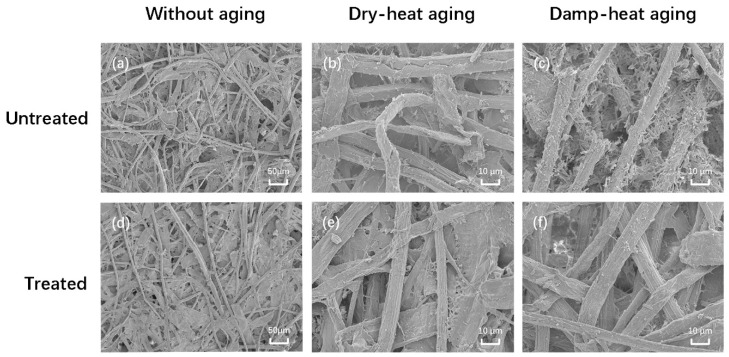
SEM images of paper samples before and after PVAm treatment. (**a**) Unaged paper before polyvinylamine treatment (**b**) Paper that has undergone dry heat aging before polyvinylamine treatment (**c**) Paper that has undergone wet heat aging before polyvinylamine treatment (**d**) Paper that has undergone dry heat aging after polyvinylamine treatment (**e**) Paper that has undergone dry heat aging after polyvinylamine treatment (**f**) Paper that has undergone wet heat aging after polyvinylamine treatment.

The SEM images of the paper samples before and after processing are depicted in Figure 2. Observations reveal that the overall morphology of the paper fibers after PVAm treatment is basically the same as before, and the basic structure of the paper fibers is not damaged. Moreover, after PVAm treatment, the paper fibers are bonded together, resulting in the elimination of numerous gaps between them. The fiber bonding becomes tighter, and it seems the surface is coated with a layer of adhesive film, enhancing the specific surface area of the fibers and the interaction force between the fibers. On a macroscopic level, this results in enhanced mechanical properties of the paper, contributing to its reinforcement. The paper before PVAm treatment experienced fiber breakage and increased fiber voids after dry heat and wet heat aging. After PVAm treatment, the treated paper is aged, the paper fibers are complete, without fracture and damage, and the bond between the fibers is relatively tight, indicating that the PVAm solution is highly effective at protecting the integrity of the paper fiber structure and greatly improves the durability of the paper.

### 3.3. Effect of Polyethylene Amine Reinforcement Solution on the Cellulose Structure in Paper

Paper cellulose is formed by the interlocking connections of crystalline and amorphous regions. The cellulose molecules in the crystalline region are arranged neatly and orderly, forming robust hydrogen bonds that enhance the paper’s robustness. In the amorphous region, cellulose molecules are arranged irregularly and have no fixed rules, resulting in weak intermolecular bonding and a small contribution to paper strength [28]. The impact of the PVAm reinforcement solution on paper’s cellulose composition can be examined using techniques like infrared spectroscopy, X-ray diffraction spectroscopy, X-ray photoelectron spectroscopy, and solid-state nuclear magnetic resonance carbon spectroscopy.

#### 3.3.1. Infrared Spectroscopy Testing

**Figure 3:**
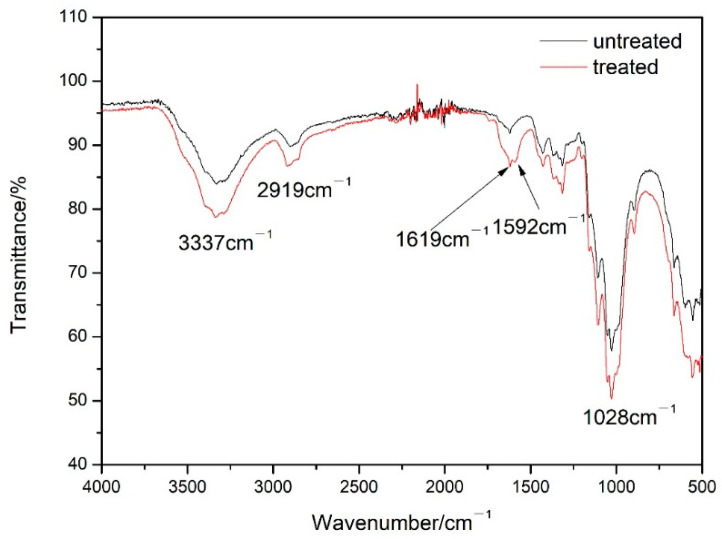
Infrared spectra of paper samples before and after PVAm reinforcement treatment.

The infrared spectra of paper samples before and after PVAm reinforcement treatment are depicted in Figure 3. Observations reveal that various types of paper exhibit distinct peaks around 3330 cm^−1^, 2900 cm^−1^, and 1027 cm^−1^, indicating the existence of -OH, -CH-, and C-O groups in the paper fibers, respectively. There was no alteration in the typical peak locations of identical paper types before and after polyethylene amine reinforcement, indicating that the reinforcement process did not affect the main functional groups of the paper. Concurrently, the paper treated with polyethylene amine produced a novel characteristic peak at 1590 cm^−1^, aligning with the typical peak of the C-N group in polyethylene amine. The value of the -OH characteristic peak at 3330 cm^−1^ in the reinforced paper decreased, indicating that groups such as -NH_2_ and -COOH in the polyethylene amine reinforcement solution combine with -OH on the cellulose molecules of the paper, generating more hydrogen bonds and enhancing the binding force between cellulose molecules, thereby reinforcing the paper. By comparing the infrared spectra of treated and untreated paper after dry heat aging (Figure S1a) and wet heat aging (Figure S1b), it can be seen that the peak position of cellulose in the aged paper is basically the same as that in the paper before aging, and no new functional groups appear, indicating that the fiber structure of the paper does not undergo significant changes after aging.

#### 3.3.2. X-ray Diffraction Testing

**Figure 4:**
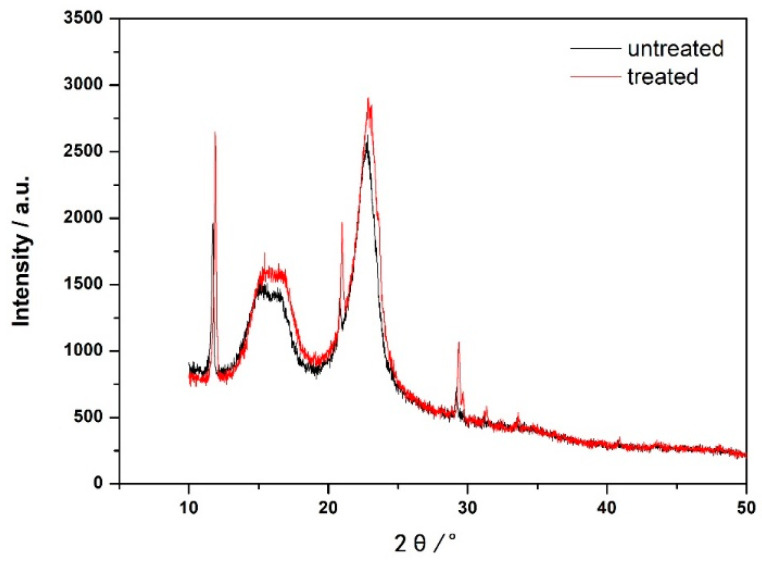
XRD patterns of paper samples before and after PVAm reinforcement treatment.

X-ray diffraction (XRD) testing was performed on paper samples to analyze the impact of PVAm addition on the hydrogen bonding strength between cellulose molecules [29]. Figure 4 illustrates that the paper’s diffraction peaks, when treated with PVAm, resemble those of the untreated paper, with the characteristic diffraction peaks of crystal plane (110) and crystal plane (002) appearing around 16.0° and 22.0°, indicating that the measured samples are all natural fiber I structures [30]. The positions of the X-ray diffraction peaks after treatment have not changed, indicating that the crystalline structure of cellulose has not changed. However, the diffraction peak intensities increased relative to similar untreated paper, signifying enhanced crystallinity post-reinforcement treatment. By comparing the XRD spectra (Figure S2) of various kinds of treated and untreated paper after dry heat aging and damp heat aging, it can be seen that the diffraction peak intensity of the paper after PVAm treatment is greater than that of the paper before treatment, regardless of whether dry heat aging or damp heat aging is used, indicating that the polyvinylamine lotion can effectively alleviate the damage to the crystal structure of paper fibers during the aging process and the aging resistance of the treated paper is stronger.

#### 3.3.3. XPS

**Figure 5:**
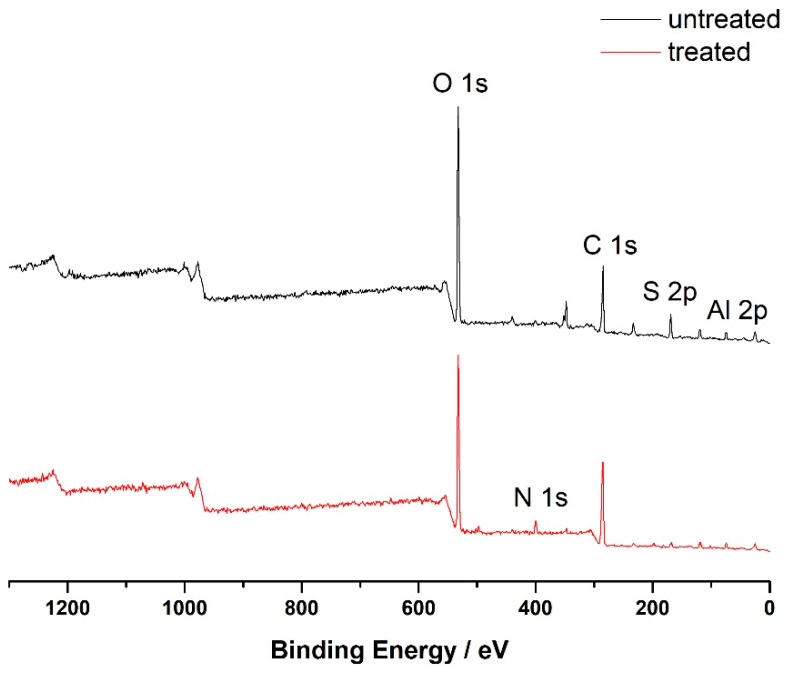
XPS spectra of paper samples before and after PVAm reinforcement treatment.

X-ray photoelectron spectroscopy (XPS) is applicable for both qualitative and quantitative analyses of elements, as well as for a better analysis of group properties on material surfaces. The C and O elements in paper fiber components can be detected and analyzed via XPS. XPS testing was performed on the paper before and after PVAm treatment, with the outcomes depicted in Figure 5.

**Figure 6:**
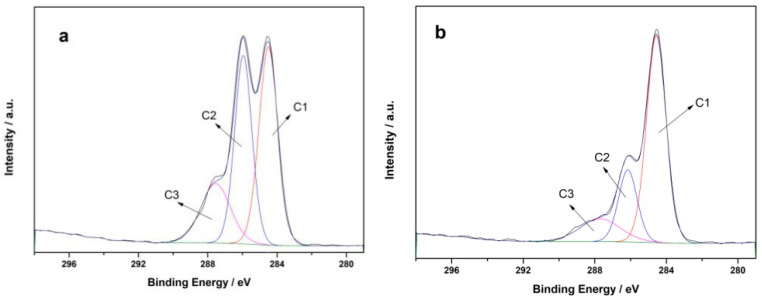
C1s XPS images of paper samples before and after PVAm treatment ((**a**) untreated paper; (**b**) processed paper).

The XPS spectrum reveals that carbon and oxygen are the primary elements in all the papers, accompanied by signals of Al and S, a result of alum water undergoing pre-acidification in the simulated paper. The paper reinforced with PVAm includes N elements because the polyethylene amine polymer contains a large number of amino groups. Due to the fact that the binding mode and amount of C element largely determine the structure and properties of fiber components, peak fitting was performed on the C1s peaks of various samples before and after treatment, as shown in Figure 6. The C1s peaks in the XPS spectra of all paper samples can be deconvolved into three different oxidation states of carbon: C1, C2, and C3, corresponding to C-C, C-O, C=O, and/or O-C-O binding energies [31]. The corresponding atomic percentage content is shown in Table 1. According to Table 1, after PVAm treatment, the contents of C-C and C=O/O-C-O in the paper increased, while the content of C-O decreased. This could be linked to the fact that the polyethylene amine polymer contains C-C and C=O groups and that the amino and carboxyl groups in the polyethylene amine combine with the hydroxyl groups in the cellulose molecule to form hydrogen bonds.

#### 3.3.4. Solid 13C NMR Nuclear Magnetic Test

**Figure 7:**
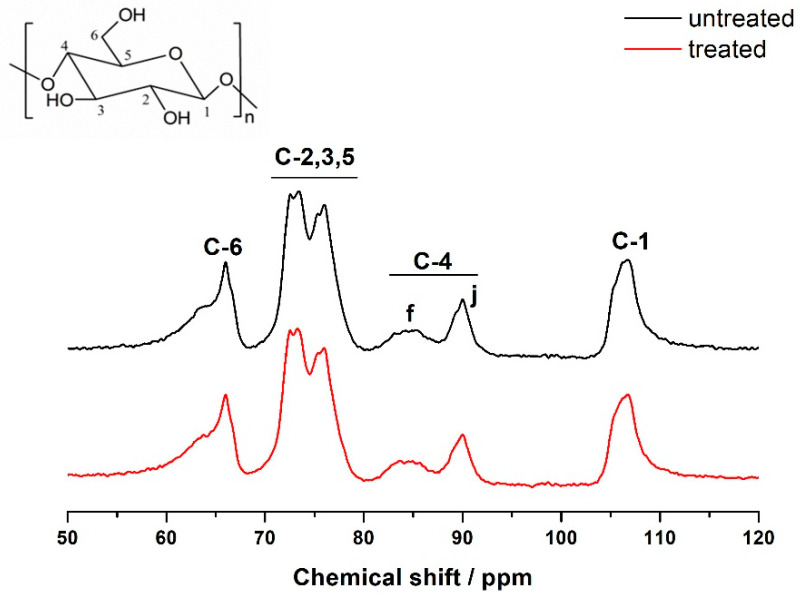
13C NMR spectra of paper samples before and after PVAm reinforcement treatment.

Solid-state nuclear magnetic resonance (13C NMR) technology is capable of directly estimating the chemical composition and structure of solid powders semi-quantitatively. This technology is sensitive to the order of the cellulose molecular chain, enabling precise analysis of the crystal structure information of cellulose. Testing the 13C NMR spectra of the paper before and after PVAm treatment enables a comparison of the alternations in its fiber crystal structure, as depicted in Figure 7. The figure illustrates that the absorption signal of paper cellulose primarily occurs in the chemical shift δ. The chemical shifts of the C element on its glucose unit at 60–110 ppm, δ 60–70 ppm, 70–80 ppm, 80–92 ppm, and 104–110 ppm correspond to C-6, C-2, 3, 5, C-4, and C-1, respectively. The C-4 signal is split into two peaks, f and j, representing the signal peaks of cellulose C-4 atoms in the amorphous and crystalline regions, located at 80–86 ppm and 86–92 ppm, respectively. f and j represent the amorphous and crystalline regions of the fiber [32]. The crystallinity of cellulose is X = Sj/(Sj + Sf), with Sj and Sf representing the peak areas of the j and f peaks, respectively [33]. According to Table 2, the crystallinity of the paper reinforced with PVAm is higher than that before reinforcement.

## 4. Conclusions

The main contribution of this study lies in exploring the use of polyethylene amine as a reinforcement agent to enhance the mechanical strength and aging resistance of fragile paper. Experimental results have demonstrated that treatment with polyethylene amine significantly improves the folding and tensile strength of the paper. Moreover, under the same aging conditions, the treated paper exhibits a slower rate of strength loss, indicating its ability to maintain its mechanical properties and enhance aging resistance. Following dry heat and damp heat aging, the paper fibers treated with polyethylene amine remain intact without breakage, suggesting the crucial role of polyvinylamine lotion in safeguarding the integrity of the paper fiber structure and improving durability. Analytical findings from infrared spectroscopy, X-ray diffraction spectroscopy, X-ray photoelectron spectroscopy, and solid-state nuclear magnetic resonance carbon spectroscopy provide evidence of enhanced hydrogen bonding between cellulose molecules and the functional groups of polyethylene amine. This further strengthens the binding force between cellulose molecules and elevates the crystallinity of the paper, ultimately reinforcing its strength. These findings highlight the significant contribution and advantages of employing polyethylene amine for reinforcing fragile paper, although potential cost implications and technical limitations should be considered before implementing this approach in practical paper manufacturing.

## Figures and Tables

**Table 1:** Relative content of various valence carbon states in paper before and after PVAm treatment.

Paper Samples	C-C (at. %)	C-O (at. %)	C=O/O-C-O (at. %)
Unprocessed	46.80	40.13	13.07
Processed	63.96	21.34	14.70

**Table 2:** Crystallinity of paper before and after PVAm treatment.

Paper Samples	Crystallinity X (%)
Unprocessed	50.48
Processed	56.35

## Data Availability

Data are contained within the article and Supplementary Materials.

## Supplementary Materials

The following supporting information can be downloaded at: https://www.mdpi.com/article/10.3390/polym16050619/s1. Figure S1. (a) FTIR spectra before and after dry heat aging; (b) FTIR spectra before and after damp heat aging. Figure S2. XRD before and after dry heat and wet heat aging.
