# Supplementary material for: Research on Strengthening Fragile Paper with Polyvinylamine
Source: Polymers (Basel). 2024 Feb 24;16(5):619. doi: 10.3390/polym16050619 (PMC10934906; doi:10.3390/polym16050619)
Supplement: Supplementary file 1 [file polymers-16-00619-s001.zip › polymers-2881159-supplementary.pdf]

polymers

MDPI

Article

Research on Strengthening Fragile Paper with Polyvinylamine

Jing Li [1], Meirong Shi [2], Yuhu Li [3,*] and Peng Fu [2,*]

[1] Shandong Museum, Jinan 250014, China; lijing9669@126.com
[2] Shaanxi Institute for the Preservation of Culture Heritage, Xi'an 710075, China; shimeirong123@163.com
[3] Engineering Research Center of Historical Cultural Heritage Conservation, Ministry of Education, School of Materials Science and Engineering, Shaanxi Normal University, Xi'an 710119, China
* Correspondence: liyuhu@snnu.edu.cn (Y.L.); fupeng@snnu.edu.cn (P.F.)

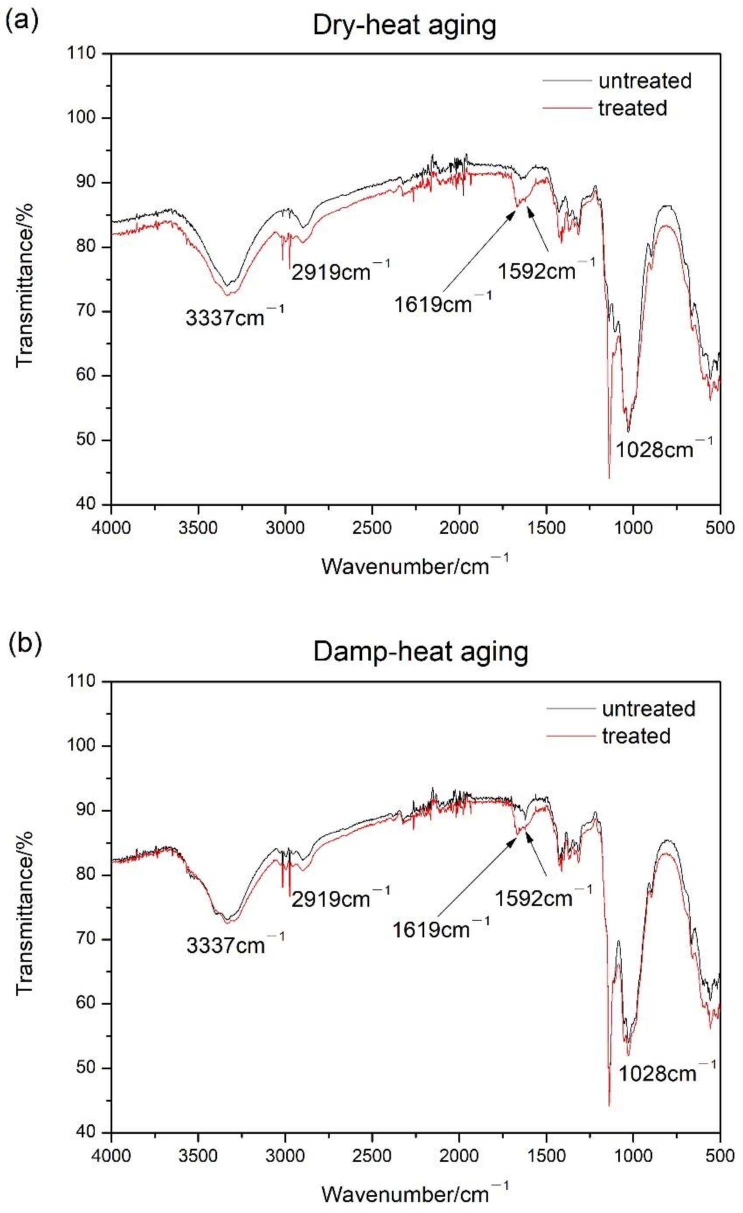

Figure S1 (a) FTIR spectra before and after dry heat aging; (b) FTIR spectra before and after damp heat aging.

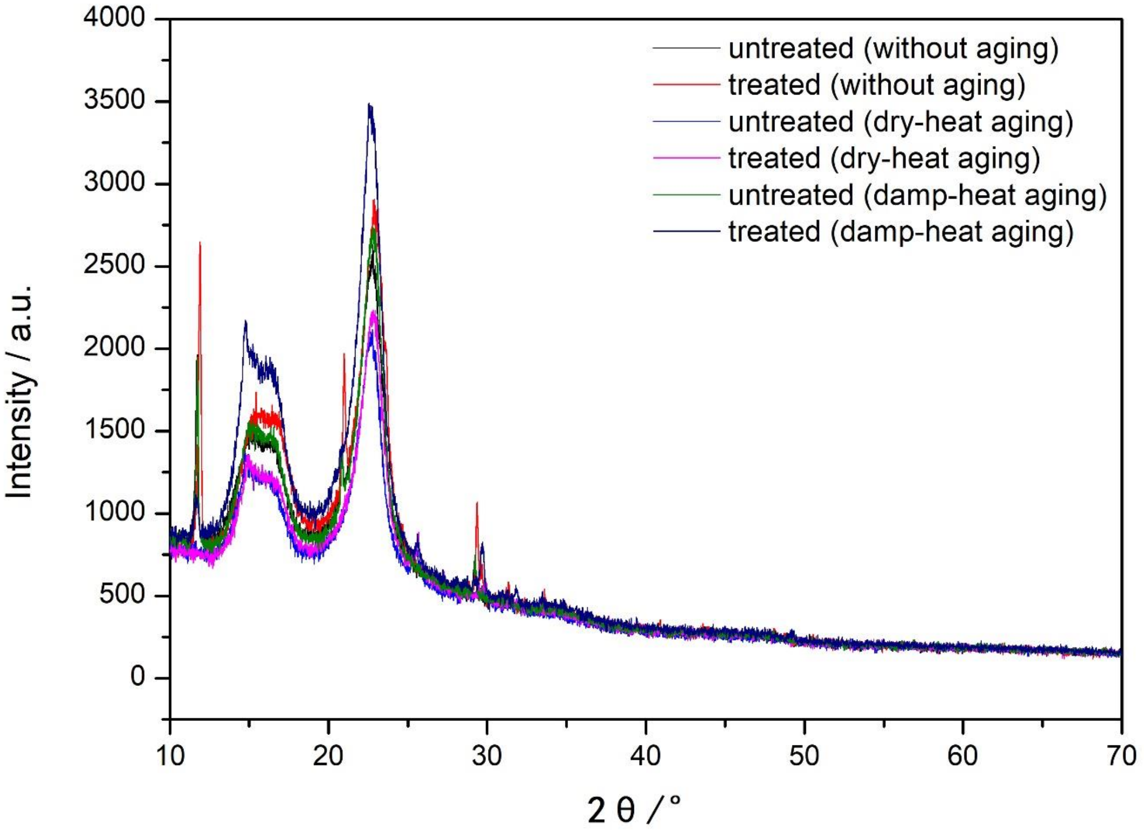

Figure S2 XRD before and after dry heat and wet heat aging
